# A named General Practitioner (GP) is associated with an increase of hospital days in a single predictor analysis: a follow-up of 15 years

**DOI:** 10.1186/s12913-023-10184-5

**Published:** 2023-10-28

**Authors:** Emmi Lautamatti, Kari J. Mattila, Sakari Suominen, Lauri Sillanmäki, Markku Sumanen

**Affiliations:** 1 Faculty of Medicine and Health Technology, Tampere University, Tampere, Finland; 2 The Wellbeing Services County of Pirkanmaa, Tampere, Finland; 3 Department of Public Health, University of Turku, Turku, Finland; 4 The Wellbeing Services County of Southwest Finland, Research Centre, Turku, Finland; 5 School of Health Sciences, University of Skövde, Skövde, Sweden; 6 University of Turku, Turku, Finland; 7 Department of Public Health, University of Helsinki, Helsinki, Finland

**Keywords:** Continuity of Care, Named GP, Hospital days, Health care services, Finnish healthcare, Register-based, Logistic regression analysis

## Abstract

**Background:**

Continuity of care constitutes the basis of primary health care services and is associated with decreased hospitalization. In Finland, accessibility to primary care and increased use of hospital services are recognized challenges for the health care system.

**Objectives:**

The aim of the study was to determine whether having a named GP is associated with hospital service use.

**Methods:**

The data are part of the Health and Social Support study (HeSSup) based on a random Finnish working-age population sample. The cohort of the study comprised participants of postal surveys in 1998 (*n* = 25,898) who returned follow-up questionnaires both in 2003 and 2012 (*n* = 11,924). Background characteristics were inquired in the questionnaires, and hospitalization was derived from national registries (Hilmo-register).

**Results:**

A named GP was reported both in 2003 and 2012 only by 34.3% of the participants. The association between hospital days and a named GP was linearly rising and statistically significant in a single predictor model. The strongest associations with hospital use were with health-related factors, and the association with a named GP was no longer significant in multinomial analysis.

**Conclusion:**

A named GP is associated with an increased use of hospital days, but in a multinomial analysis the association disappeared. Health related factors showed the strongest association with hospital days. From the perspective of the on-going Finnish health and social services reform, continuity of care should be emphasized.

## Background

Continuity of care is a key factor for health care organizations. It induces multiple benefits, such as decreased mortality and use of hospital service in combination with improved patient satisfaction and cost-effectiveness [1–5]. Accessibility and availability are required in order to implement continuity in primary health care services [6]. Regular contact with a primary care general practitioner (GP) reduces emergency and other hospital admissions [7]. Barker et al. [7] noticed an inverse association between continuity of care and rate of unexpected hospitalization. Moreover, preventable hospitalizations seemed to be avoided more easily among the population with continuity of care [1, 7]. In a separate study, the length of the individual hospital stay was also shorter when continuity during hospital care was implemented [8].

Continuity of care can be roughly divided into three dimensions: interpersonal, managemental, and informational continuity of care [9]. In international studies, continuity of care is often understood as an interpersonal patient-doctor relationship. The population’s perspective as health care service users should also be noted [10]. As Baker et al. [2] have suggested, continuity of care should be considered as a subjective rather than an objective factor.

The Finnish health care system consists of primary health care, secondary care (hospitals) and preventive care [11]. Primary health care is the basis of health care services in Finland [12]. It provides preventive health care in combination with medical and rehabilitation services. The services are organized in health centres, where doctors, nurses, and physiotherapists work as a multidisciplinary team. There is a long tradition of having a named and assigned GP for every Finn. The named GP is the primary health care provider, who is allocated to the patient by the local primary health care organization. The named GP and the established personal doctor-patient relationship creates continuity of care, around which municipalities organize the production of primary health care services. Naturally, for continuity of care to implement, also accessibility to health care services should be enabled. In previous studies multiple benefits of continuity of care seem to be mediated to the population by named GPs [13–15].

In Finland in 1972, the municipalities were by legislation obliged to organize both primary and secondary health care services for their population [16]. The health care system, where municipalities had the responsibility to organize and finance health care services was unique in the world. In the beginning of the year 2023 the health care legislation was renewed. The responsibility of organizing the health care services is now allocated to welfare districts. The government finances the service production. [11] To access secondary care, such as hospital services in non-urgent matters, the patient needs a referral from primary health care. Usually, municipalities purchase secondary care services from hospital districts or other providers. Doctors in privately or publicly funded occupational health care and private independent health units can offer health care services, including referral to secondary care. There are national guidelines to assure equal referral policies [17].

After the economic depression in the 1990s, the system with in-built continuity of care (a named GP at the health centre) became endangered in Finland. Lack of both financial and human resources in primary health care forced municipalities to re-organize the production of services, and continuity of care was no longer a key factor [18]. Both continuity of care and accessibility to services decreased [19].

Since the 1990s, multiple different personal doctor schemes have been implemented in Finnish primary care. Inadequate resources, oversized catchment areas, and the physicians’ unspecified working hours have finally resulted in a nationwide crisis. A lack of named GPs and continuity of care have come to define Finnish primary care [20, 21].

In Finland, all use of social and health care services is registered in the Finnish national Care Register for Health Care (Hilmo-register). The register aggregates information and produces statistics among other things on population service use, accessibility to services, population’s health problems, epidemics, illness prevention and health promotion services [22]. Health care professionals in health centres and university, central or local district hospitals register the visits by making entries into the electronic health records. The Finnish Institute of Health and Welfare uses the data to generate national statistics of continuity of care, access, and use of the services, which are used for service monitoring and development [22–24]. The data of the present study are from the Finnish Hospital Discharge Register, which constitutes an essential part of the Hilmo-register’s data on hospital use with a coverage of 95% of hospital discharges in Finland [24].

In Finland, the premises for establishing continuity of care have deteriorated in recent decades. The contact between the named GP and the patient have constituted the basis for continuity of care all these years despite lack of resources. The aim of the study is to create knowledge on the state of continuity of care in Finland and the associations of social and health-related factors with Hilmo-registered hospital use. We propose that a named GP still can mediate the benefits of continuity of care to the population in a health care environment already affected by fragmented continuity. Is continuity of care operationalized via a named GP’s association with the use of hospital services?

## Methods

**Fig. 1:**
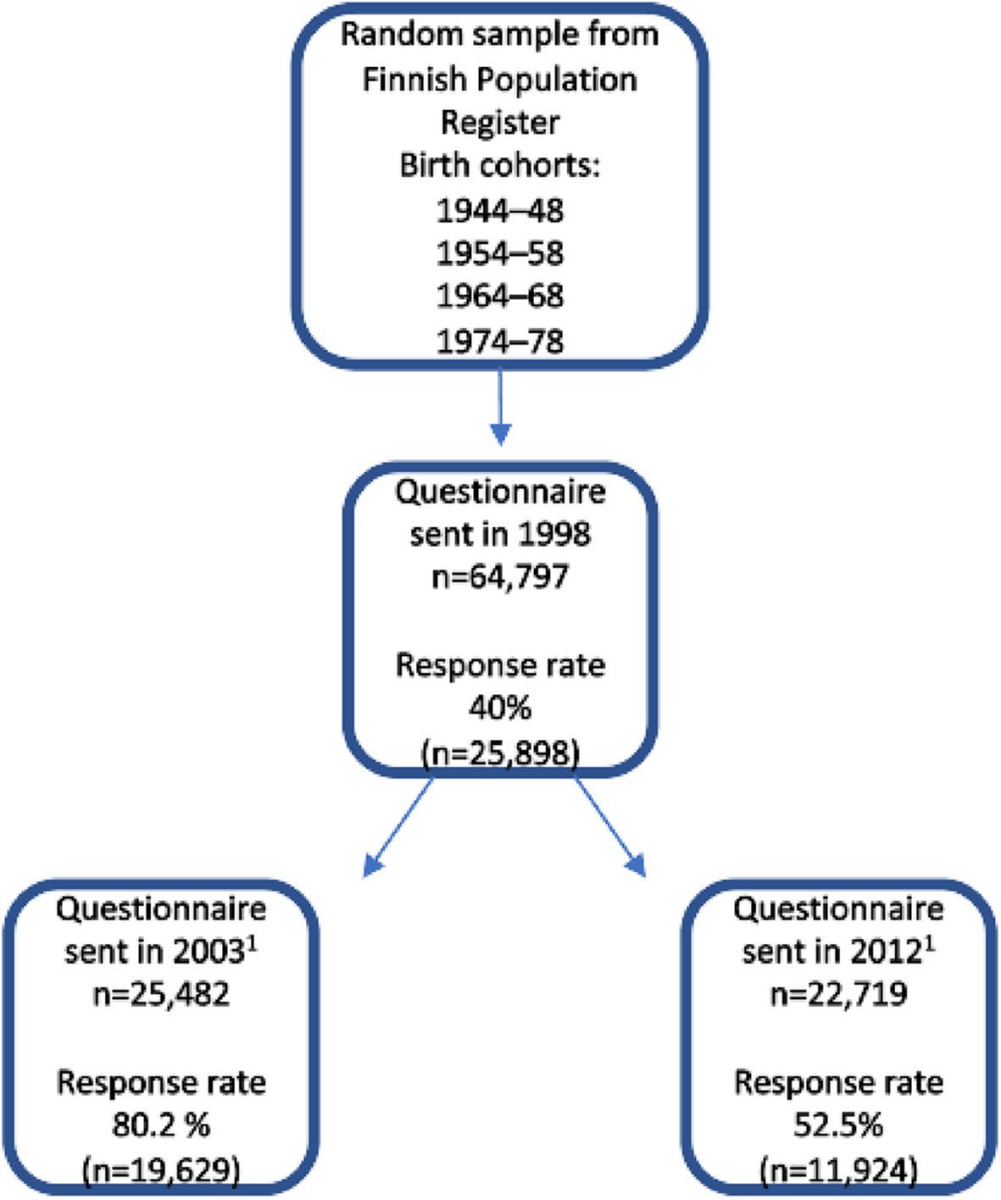
The procedure of forming the data for the HeSSup study in Finland. ^1^) Participants who were deceased, had emigrated**,** or had declined disclosure of their address from the Finnish Population Register were excluded

The participants and data originated from the Health and Social Support (HeSSup) study [25, 26]. A random sample of 64,797 working-aged individuals drawn from the Finnish Population Register comprised four birth cohorts: 1944–1948, 1954–1958, 1964–1968, and 1974–1978. The response rate in 1998 was 40% (*n* = 25,898). The survey was repeated in 2003 (response rate 80.2%) and 2012 (response rate 54.5%) with the respondents of the 1998 survey. Only participants having responded every year were included in the study. Individuals who had emigrated, declined disclosure of their address from the Finnish Population Register, or died were excluded. The data can be considered representative of the Finnish population, particularly in relation to morbidity [25]. A careful non-response analysis in 1998 indicated that there were no health-related factors disputing the comparability of respondents and non-respondents [26] (Fig. 1).

The participants of the HeSSup study had given written consent to combine the reported information with the national registries, including the mortality records of the Finnish Population Register, Cancer Registry, the medical records of the Social Insurance Institution of Finland, and the Hilmo-register.

### Outcome variables

The number of hospital days of the HeSSup study participants were gathered from the Hilmo-register and linked with the reported information from the surveys. Hospital days with pregnancy-related diagnoses were excluded from the Hilmo-register data to establish a public health-based perspective of the association of hospital service use and morbidity.

**Fig. 2:**
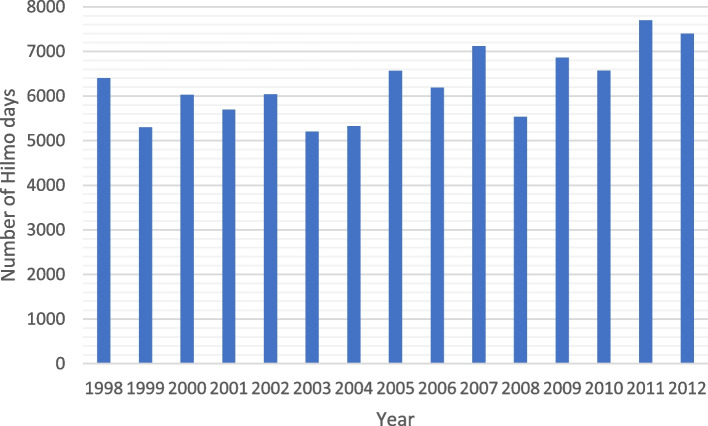
Distribution of Hilmo-registered hospital days in 1998–2012 among the participants of the HeSSup study in Finland. Pregnancy related hospital days excluded

The outcome variable of hospital days was calculated as a sum of hospital days from 1998 to 2012. The distribution of the days per year is seen in Fig. 2. The sum of the days was categorized into three groups – < 1 days, 1–3 days, and > 3 days – based, respectively, on the median of the variable for each subject.

### Explanatory variables

#### Social background

All social background factors were derived from the 2012 survey data. Age was categorized into classes according to the four birth cohorts (1974–1978, 1964–1968, 1954–1958, and 1944–1948). The youngest cohort included 20.7% (*n* = 2470) of the respondents, the 1964–1968 cohort 20.5% (*n* = 2440), the 1954–58 birth cohort 26.7% (*n* = 3185) and the oldest birth cohort 32.1% (*n* = 3829) of respondents. Participants with a degree from a university, college, or polytechnic were considered as having higher education, whereas the remainder were considered to have a lower level of education (Table 1).

**Table 1:** Characteristics of participants of the HeSSup study in 1998, 2003 and 2012 (*n* = 11,924) categorized by hospital days derived from Hilmo-register data in Finland. Hospital days with pregnancy-related diagnoses excluded

	Hospital days		Hospital days		Hospital days			
Characteristics	0		1–3		> 3		Total	
	n	%	n	%	n	%	n	%
VARIABLES								
Gender								
Male	1581	35.7	1264	28.6	1582	35.7	4427	100
Female	2633	35.1	2325	31.0	2539	33.9	7497	100
Birth cohort								
1974–78	1246	50.5	733	29.7	491	19.9	2470	100
1964–68	975	40.0	840	34.4	625	25.6	2440	100
1954–58	1029	32.3	970	30.5	1186	37.2	3185	100
1944–48	964	25.2	1046	27.3	1819	47.5	3829	100
Native language								
Finnish	3733	34.9	3218	30.1	3746	35.0	10,697	100
Swedish	481	39.2	371	30.2	375	30.6	1227	100
Education								
Lower	2486	31.2	2378	29.9	3096	38.9	7960	100
Higher	1712	44.0	1186	30.5	989	25.4	3887	100
HEALTH STATUS								
Chronic disease								
No	1953	51.3	1197	31.4	658	17.3	3808	100
Yes	2261	27.9	2392	29.5	3463	42.7	8116	100
Health status								
Good	4114	36.8	3469	31.0	3598	32.2	11,181	100
Poor	77	11.5	102	15.3	489	73.2	668	100
Smoking								
No	3141	34.3	2825	30.9	3182	34.8	9148	100
Yes	466	30.6	458	30.1	600	39.4	1524	100
Obesity								
BMI < 25 kg/m^2^	2083	40.6	1558	30.4	1486	29.0	5127	100
BMI ≥ 25 kg/m^2^	2079	31.2	1995	30.0	2581	38.8	6655	100
Functional limitations								
NYHA 0–1	4012	37.5	3336	31.2	3354	31.3	10,702	100
NYHA 2–4	186	16.1	242	20.9	728	63.0	1156	100
Depressive mood								
BDI < 19	4047	36.0	3420	30.4	3788	33.7	11,255	100
BDI ≥ 19	122	23.0	137	25.8	272	51.2	531	100
Named GP^a^								
No 2003 nor 2012	1195	36.4	1028	31.3	1064	32.4	3287	100
2003 or 2012	1440	35.6	1152	28.5	1453	35.9	4045	100
2003 and 2012	1378	33.7	1256	30.7	1459	35.6	4093	100

^a^Named GP measured longitudinally in 2003 and 2012

#### Health and health behaviour

Chronic diseases were inquired in the 2012 survey by the question “Has a physician ever said that you have or have had…” followed by a list of 32 individual diseases as response alternatives. The participant marked the absence or presence of any disease. Boxes left blank were categorized as a negative response. In total, 26 of the 32 diseases reported were categorized as chronic according to the National Institute for Health and Care Excellence (NICE) guidelines [27]. (Table 2) Participants in the “Chronic disease” group reported one or more chronic diseases, while the rest formed the “No chronic disease” group (Table 1). The share of participants who reported chronic diseases were evenly distributed according to all the three classes of the factor ‘Named GP’ (Table 3).

**Table 2:** The chronic diseases reported by the participants of the 2012 survey of the Health and Social Support (HeSSup) Study (*n* = 11,924) in the order of reported prevalence

	Reported diseases	
	n	%
Arthrosis	2598	21.8
Migraine	2485	20.8
Depression	2026	17.0
Hypertension	1967	16.5
Long term bronchitis/emphysema	1132	9.5
Asthma	1016	8.5
Diabetes	878	7.4
Panic attack disorder	820	6.9
Cataract or glaucoma	772	6.5
Cancer	708	5.9
Atrial fibrillation/flutter	484	4.1
Other mental disorder	478	4.0
Rheumatoid arthrosis	407	3.4
Fibromyalgia	395	3.3
Other neurological disease	382	3.2
Transient Ischemic Attack	368	3.1
Angina pectoris	328	2.8
Kidney disease	249	2.1
Myocardial infarction	242	2.0
Coeliac disease	225	1.9
Eating disorder	204	1.7
Liver disease	190	1.6
Epilepsy	189	1.6
Brain injury	157	1.3
Stroke	133	1.1

**Table 3:** Distribution of the reported chronic diseases according to the factor ‘Named GP’

	No named GP		Named GP		Named GP		
	2003 nor 2012		2003 or 2012		2003 and 2012		
	(*n* = 3287)		(*n* = 4045)		(*n* = 4093)		
	n	%	n	%	n	%	*p*
Chronic diseases							< 0.001
No	1198	33.2	1245	34.5	1167	32.3	
Yes	2089	26.7	2800	35.8	2926	37.4	

The Named GP factor is calculated from the 2003 and 2012 surveys of the Health and Social Support (HeSSup) Study in Finland (*n* = 11,924). Considering reports of the factor Chronic diseases (*n* = 11,425), the participant was categorized to "Yes" if they had reported one or more chronic disease in the 2012 survey

Perceived health was inquired with a five-point Likert scale in the 2012 survey. The options “good” and “fairly good” were brought together to form the class good. The remaining options were considered poor based on the argumentation that the respondent did not consider her/his health as good. Smoking was dichotomised into the groups “Yes” when the respondent reported being a smoker at the time of the survey and otherwise “No”. Body Mass Index (BMI) was cut into two categories with a cut-point of 25 kg/m^2^ [28]. The New York Heart Association (NYHA) classification describes the functional limitations of the respondent. All-cause exertional shortness of breath reported in the survey was scored according to the NYHA classification [29]. The NYHA scores were categorized into 0–1 and 2–4, with 0 meaning no symptoms in exercise or physical activity. Beck’s Depression Inventory (BDI) was used to assess the participants’ possible depressive mood. Values < 19 were considered as normal or mildly depressive mood, while participants with values ≥ 19 were considered to have moderate or difficult depression [30] (Table 1).

Participants reporting a named GP were determined according to the 2003 and 2012 survey data with the single question: “Do you have an assigned and named GP at your local health centre?” The options “Yes” and “No” indicated the existence or lack of a named GP and thus the continuity – or lack thereof – of care. The answer was categorized into three classes based on the participants’ report: no named GP in either of the years (“Neither 2003 nor 2012”), a named GP in one of the years (“2003 or 2012”), and a named GP in both years (“2003 and 2012”) (Table 1).

The associations between the outcome variable and explanatory variables were first studied in single predictor logistic regression analyses according to the variables on health and health behaviour. An additional logistic regression analysis included perceived health and chronic diseases as covariates. The interaction effect of hospital use, and a named GP was analysed as well. The limit for statistically significant results was set at *p* < 0.05.

Statistical analyses were conducted with IBM SPSS Statistics, version 27 (IBM Corp. Armonk, NY, USA, 2020) and SAS software 9.4 TS1M5 (SAS Institute Inc., Cary, NC, USA, 2016).

## Results

A named GP was reported in both 2003 and 2012 only by 34.3% (*n* = 4093) of the participants. The respondents with no named GP in 2003 nor 2012 had more often no hospital service use (36.4% vs 33.7%) than participants with a named GP. Correspondently participants with a named GP in both 2003 and 2012 had more often more than three hospitalization days compared to participants with no named GP (35.6% vs 32.4%). The differences were statistically significant (*p* < 0.001). Participants with higher age (47.5% vs 37.2%) or at least one reported chronic disease (42.7% vs 17.3%) had also used hospital services more often than younger or healthier participants (*p* < 0.001, *p* < 0.001). (Table 1).

The total count of the Hilmo-registered hospital days varied between 5205 (in 2003) and 7699 (in 2011) days. Besides, an increasing trend in the number of hospital days was noted (Fig. 2).

The association between hospital days and continuity of care was linear in the single predictor analysis, reaching statistical significance for the group with more than 3 days for participants with a named GP in both 2003 and 2012 (OR 1.19; 95% CI 1.07–1.33) (Table 4).

**Table 4:** Single predictor logistic regression analyses according to hospital days derived from the Hilmo-register among the participants of the 1998, 2003, and 2012 HeSSup surveys in Finland.^a^The outcome variable is register-based hospital days categorized into three groups: 0 (reference), 1–3, and more than 3 hospital days. No covariates were included in the models

	Hospital days		Hospital days	
Characteristics	1–3		> 3	
	OR (95% Cl)	*p*	OR (95% Cl)	*p*
VARIABLES				
Gender		0.036		0.413
Male	1		1	
Female	1.04 (1.01–1.21)		0.96 (0.88–1.05)	
Birth cohort				
1974–78	1		1	
1964–68	1.46 (1.29–1.67)	< 0.001	1.63 (1.41–1.88)	< 0.001
1954–58	1.60 (1.41–1.82)	< 0.001	2.93 (2.56–3.34)	< 0.001
1944–48	1.84 (1.63–2.10)	< 0.001	4.79 (4.20–5.46)	< 0.001
Education		< 0.001		< 0.001
Higher	1		1	
Lower	1.38 (1.26–1.52)		2.16 (1.96–2.37)	
HEALTH STATUS				
Chronic disease		< 0.001		< 0.001
No	1		1	
Yes	1.73 (1.57–1.89)		4.55 (4.10–5.04)	
Health status		0.003		< 0.001
Good	1		1	
Poor	1.57 (1.17–2.12)		7.26 (5.69–9.27)	
Smoking		0.015		< 0.001
No	1		1	
Yes	1.19 (1.03–1.36)		1.38 (1.21–1.57)	
Obesity		< 0.001		< 0.001
BMI < 25 kg/m^2^	1		1	
BMI ≥ 25 kg/m^2^	1.28 (1.17–1.40)		1.74 (1.59–1.90)	
Functional limitations		< 0.001		< 0.001
NYHA 0–1	1		1	
NYHA 2–4	1.57 (1.29–1.91)		4.68 (3.96–5.54)	
Depressive mood		0.025		< 0.001
BDI < 19	1		1	
BDI ≥ 19	1.33 (1.04–1.70)		2.38 (1.92–2.96)	
REGIONAL SERVICE CHARACTERISTICS				
Named GP^b^				
Neither 2003 nor 2012	1		1	
2003 or 2012	0.93 (0.83–1.04)	0.211	1.13 (1.02–1.27)	0.026
2003 and 2012	1.06 (0.95–1.19)	0.316	1.19 (1.07–1.33)	0.002

^a^Pregnancy related hospital days excluded

^b^Named GP measured longitudinally in 2003 and 2012

In single predictor logistic regression models, the strongest association with more than three hospital days was with perceived health (OR 7.26; 95% CI 5.69–9.27). Also, reported chronic diseases and NYHA score 2–4 increased the odds for multiple hospital days twofold to fourfold (*p* < 0.001). Higher age increased the odds for hospitalization more than fourfold compared with the younger participants (*p* < 0.001) (Table 4).

In an additional logistic regression analysis, perceived health and reported chronic diseases were included as covariates. Higher age and NYHA score 2–4 were most strongly associated with more than three hospital days by more than doubling the odds ratio (*p* < 0.001). The association with a named GP was no longer statistically significant (Table 5).

**Table 5:** Logistic regression analyses according to hospital days derived from the Hilmo-register days among the participants of the 1998, 2003, and 2012 HeSSup surveys in Finland.^a^The outcome variable is register-based hospital days categorized into three groups: 0 (reference), 1–3, and more than 3 hospital days. All factors included in the same analysis. Covariates included in the model are perceived health and reported chronic diseases

	Hospital days		Hospital days	
Characteristics	1–3		> 3	
	OR (95% Cl)	*p*	OR (95% Cl)	*p*
VARIABLES				
Gender		0.589		0.003
Male	1		1	
Female	1.03 (0.93–1.13)		0.84 (0.76–0.92)	
Birth cohort				
1974–78	1		1	
1964–68	1.44 (1.26–1.64)	< 0.001	1.52 (1.31–1.76)	< 0.001
1954–58	1.50 (1.32–1.71)	< 0.001	2.32 (2.02–2.67)	< 0.001
1944–48	1.63 (1.43–1.86)	< 0.001	3.42 (2.99–3.92)	< 0.001
Education		< 0.001		< 0.001
Lower	1		1	
Higher	1.33 (1.21–1.46)		1.86 (1.68–2.05)	
HEALTH STATUS				
Smoking		0.028		0.001
No	1		1	
Yes	1.17 (1.02–1.34)		1.26 (1.10–1.44)	
Obesity		< 0.001		< 0.001
BMI < 25 kg/m^2^	1		1	
BMI ≥ 25 kg/m^2^	1.23 (1.13–1.35)		1.53 (1.39–1.68)	
Functional limitations		0.004		< 0.001
NYHA 0–1	1		1	
NYHA 2–4	1.34 (1.10–1.65)		2.73 (2.28–3.26)	
Depressive mood		0.341		0.167
BDI < 19	1		1	
BDI ≥ 19	1.13 (0.88–1.46)		1.18 (0.93–1.50)	
REGIONAL SERVICE CHARACTERISTICS				
Named GP^b^				
Neither 2003 nor 2012	1		1	
2003 or 2012	0.90 (0.80–1.01)	0.068	1.04 (0.92–1.16)	0.556
2003 and 2012	1.01 (0.90–1.13)	0.912	1.04 (0.92–1.17)	0.539

^a^Pregnancy related hospital days excluded

^b^Named GP measured longitudinally in 2003 and 2012

## Discussion

Only a minority of the participants reported a named GP at both observation points. A named GP represents continuity of care in the present study likewise in general Finnish circumstances [21]. In the study, a named GP – and thus continuity of care – was associated with an increased use of more than 3 hospital days, but in a multinomial analysis the association disappeared.

The association with > 3 hospital days was strongest among the population with chronic disease, functional limitations, obesity, or higher age. The population, who has chronic diseases or lower functional abilities is in greater risk of hospitalization. At the same time, they are the part of the population, who benefits from continuity of care the most [31]. It is possible that having a named GP increases accessibility to health care services, which could also be one of the mechanisms mediating the benefits of continuity of care to the population [1, 2, 6, 7].

Continuity of care decreases the use of hospital services [1], but it is not sufficiently achieved only by having a named GP. A lack of resources has driven Finnish primary care into a crisis with fragmented continuity of care [20]. The finding can be interpreted as a failure of Finnish primary care to produce continuity, which then fails to decrease use of hospital service. Regardless, a named GP increases the population’s satisfaction with health care services [14].

In a situation of decreased accessibility, fragmented continuity of care and lack of resources the named GP increases accessibility of health care services [15]. The contribution of the named GP to operationalize continuity of care is amplified in the present study. The named GP enables access to hospital services, although health-related factors come first. In international studies continuity of care decreases use of hospital services in an environment with adequate accessibility to the services [1, 2]. At the moment in Finland the premises to mediate the benefits of continuity of care to the population are not enabled. Anyhow it is still possible, that named GPs provide care to the most ill patients, which can be seen as an attempt to maintain high quality of the health care.

A named GP in Finland is assigned to the population by the local primary health care organizations. Thus, it represents a managemental procedure to provide continuity of care. However, it is possible that the procedure is not effective enough as neither premises nor benefits of continuity of care are achieved. Lack of human and financial resources in primary health care contributes to population’s unmet needs for service, which jeopardizes the quality of the total health care. Primary health care should be strengthened and new methods to ensure continuity of care should be developed.

The study has multiple strengths but carries also limitations. The data are from 1998–2012 but the concept of continuity is sustainable and benefits from an observation period of 15 years of both self-reported and register follow-up data [1, 2]. Additionally, no profound reform of the Finnish health care has taken place during that period which could have changed the common understanding and interpretation of the concept of continuity of care. The Hilmo-register provides statistics on hospital days, and the data have been shown to be reliable and accurate [24]. The results show associations between the use of hospital service and health risks that are universally known to increase the use of health care, which supports the present findings. The data from the HeSSup study can be generalized to Finnish population [25].

Continuity of care was assessed with a single question presented to the participants of the HeSSup survey, which is a major limitation of the study, enabling reporting bias. Although the participants reported a named GP twice, this does not guarantee that the identity of the GP had not been changed. Nevertheless, continuity of care has been shown to have a good face validity and should be determined subjectively, not objectively [2]. In Finland, a named and assigned GP is a concept representing continuity of care to the population. In previous studies, the concept has been shown to associate positively with satisfaction and the use of health care services (14, 15), which supports the adequateness of the use of only a single question.

Despite efforts to build effective and high-quality primary health care in Finland, the system is in crisis. True continuity of care is not reached, and a named GP does not seem to decrease use of hospital services. Thus, the findings can be interpreted cautiously as indicating that particularly patients with a named GP more probably receive the services needed, whereas this is questionable for the rest. As forthcoming study topics, the association of a named GP with use of preventive services, emergency room visits, length of hospital stays, or mortality is suggested. The high quality of the Finnish health care system is jeopardized. In the environment of an on-going reform in Finland aiming at integration of health care and social service the resources for primary health care should be secured thus, enabling true continuity of care.

## Conclusions

Continuity of care is widely known to prevent and decrease hospital use, although in Finland these benefits are not mediated to the population. The named GP is associated with an increase of hospital days, but in a multinomial analysis the association disappeared. Health related factors showed the strongest association with hospital days. From the perspective of the on-going Finnish health and social services reform, continuity of care should be emphasized.

## Data Availability

All data analysed during this study are included in this published article. The HeSSup study owns the data and publishing the data more precisely demands consent from the HeSSup study group.

## Abbreviations

BDI: Beck’s Depression Inventory
BMI: Body Mass Index
CI: Confidence Interval
GP: General Practitioner
HeSSup: Health and Social Support study
Hilmo: Finnish National Care Register for Health Care
NICE: National Institute for Health and Care Excellence
NYHA: New York Heart Association
OR: Odds Ratio
SPSS: IBM SPSS Statistics program
